# Minimally Invasive Treatment of Sporadic Burkitt's Lymphoma Causing Ileocaecal Invagination

**DOI:** 10.1155/2018/6265182

**Published:** 2018-04-30

**Authors:** Paolo Panaccio, Michele Fiordaliso, Domenica Testa, Lorenzo Mazzola, Mariangela Battilana, Roberto Cotellese, Federico Selvaggi

**Affiliations:** ^1^Department of Medical and Oral Sciences and Biotechnologies, “G. d'Annunzio” University, Chieti, Italy; ^2^General Surgery Unit, Renzetti Hospital, Lanciano, Italy

## Abstract

**Introduction:**

Primary NHL (non-Hodgkin lymphoma) of the colon represents only 0.2% to 1.2% of all colonic malignancies. Burkitt's lymphoma (BL) is usually a disease reported in children and young people, most of them associated with EBV or HIV infection. We describe a rare case of intestinal obstruction due to sporadic Burkitt's lymphoma causing ileocaecal invagination explaining our experience *Methods*. A 31-year-old man presented with diffuse colic pain and weight loss. Clinical examination revealed an abdominal distension with pain in the right iliac fossa. Colonoscopy documented a caecal large lesion with ulcerated mucosa. Computed tomography (CT) have shown a 60 × 50 mm right colic parietal lesion with signs of ileocolic intussusception.

**Results:**

Laparoscopic right hemicolectomy was performed. Postoperative period was uneventful. CD20+ high-grade B-cell Burkitt's lymphoma was confirmed by immunohistochemistry (CD20+, CD79+, and CD10+) and FISH test (t (8;14) (q24; q32). The patient was subsequently treated with adjuvant combination chemotherapy (Hyper-CVAD) and is alive and disease-free at 8 months follow-up.

**Discussion:**

Adult sporadic Burkitt's lymphoma (BL) causing intestinal obstruction due to ileocaecal intussusception is an extremely rare occurrence and a diagnostic dilemma. Despite the surgical approach is selected based on patient's conditions and surgeon's expertise, minimally invasive method could be preferred.

## 1. Introduction

Burkitt's lymphoma (BL) is usually viewed in children and young adults and rarely in middle-aged adults. BL is caused by chromosomal translocation and deregulation of the c-MYC oncogene; in nonendemic BL regions, it manifests highly aggressive and fast-growing B-cell malignancy. BL cause 5% of all bowel intussusception and 1% of all bowel obstruction. The localization of BL in the ileocaecal region is rare, especially in adults [1]. Intussusception in adults is often associated with organic pathology. We describe a case of BL-related ileocaecal intussusception in a young man successfully managed by laparoscopic-assisted surgery.

## 2. Case Report

**Figure 1:**
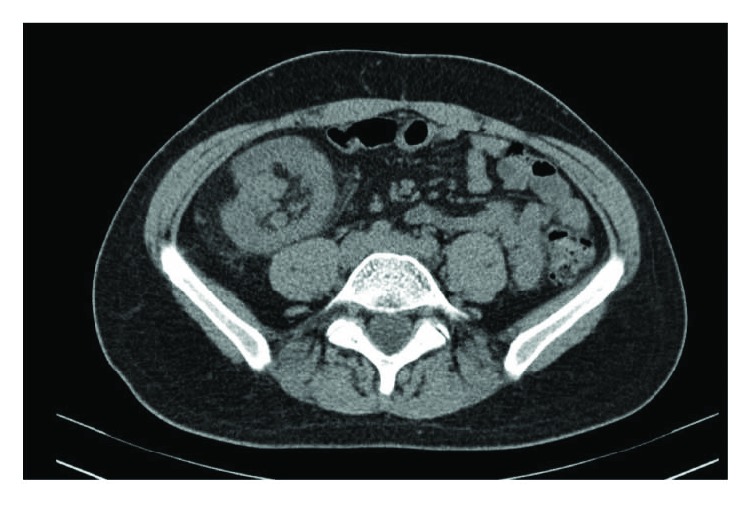
CT showed a hyperdense 60 × 50 mm right colic parietal lesion, signs of ileocolic intussusception with adjacent lymphadenopathy.

**Figure 2:**
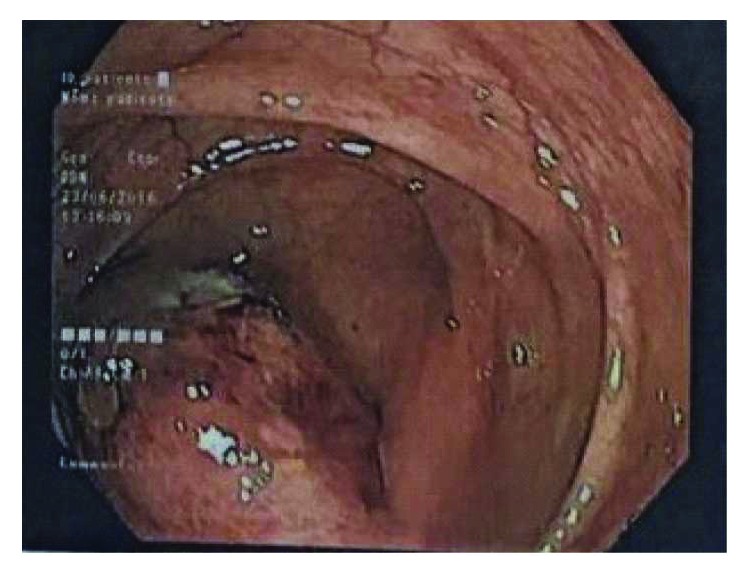
Flexible colonoscopy documented a caecal large submucosal lesion with ulcerated mucosa.

A 31-year-old man without previous medical history, except for a posttraumatic pneumothorax, presented to our emergency department with a 2-week history of diffuse colic pain and weight loss. Physical examination showed abdominal distension, a localized pain, and a palpable mass in the right lower quadrant. Laboratory studies were normal (WBC 4.99 × 10^3^/*μ*L; HGB 9.9 g/dL; HIV-EBV tests were negative; CEA and CA 19–9 were negative). A computed tomography (CT) of the abdomen showed a three-layered structure giving the characteristic target-shaped appearance in the ascending colon. Moreover, the CT showed a hyperdense 60 × 50 mm right colic parietal lesion, signs of ileocolic intussusception with adjacent lymphadenopathy measuring 20 mm (Figure 1). Flexible colonoscopy documented a caecal large submucosal lesion with ulcerated mucosa (Figure 2).

**Figure 3:**
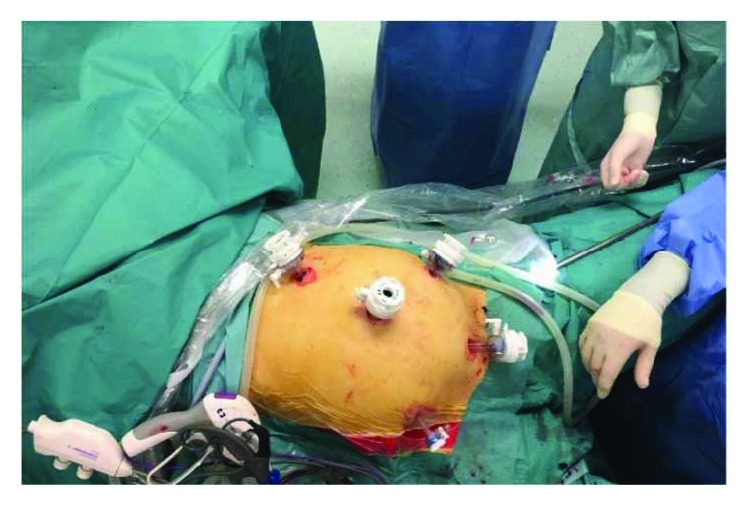
Trocar's disposition. Laparoscopic right hemicolectomy.

**Figure 4:**
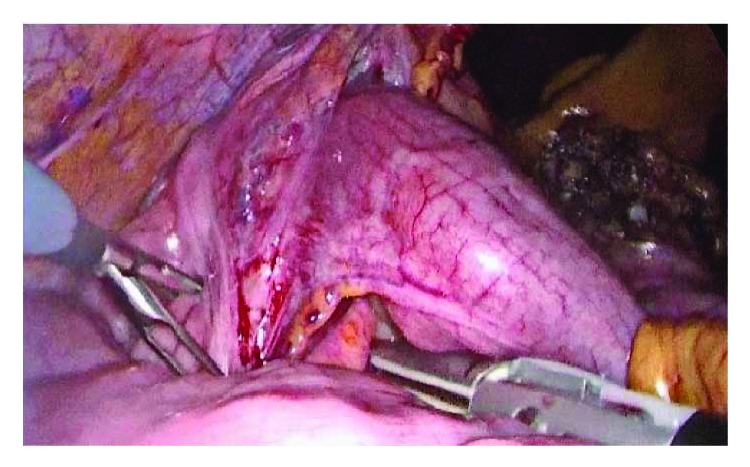
Intraoperative view of ileocolic intussusception.

**Figure 5:**
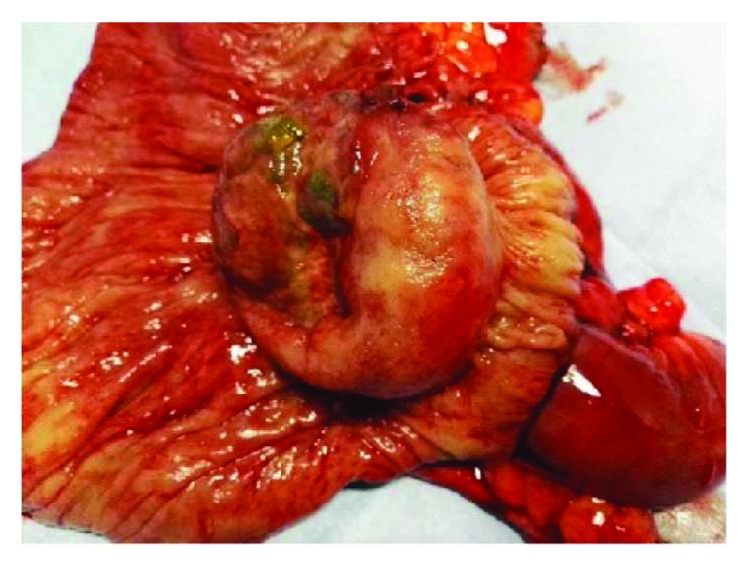
Surgical specimen after opening of the colon with appearance of tumor at the ileocaecal valve.

Laparoscopic exploration was performed: the 5-port method is generally used. A 10 mm trocar is inserted 1 cm below the umbilicus as an observation port. Another 12 mm trocar is introduced in the left flank 3 cm above the upper iliac crest as a major hand port. A 5 mm trocar is then inserted in the epigastrium (1 cm below xiphoid process). Two accessory trocars (5 mm and 10 mm) were positioned, respectively, in the right iliac fossa and suprapubic region. The surgical table is declined about 10–15° into the Trendelenburg position with a slight rotation on the left flank. The surgeon was between the patient's legs, and the camera operator/assistant was on the left side (Figure 3). Ileocolic intussusception causing occlusive status with multiple lymphadenopathies along the ileocaecal artery was observed intraoperatively (Figure 4). Laparoscopic right hemicolectomy was performed following strictly oncologic principles with ileocolic, right colic, and right branch of middle colic artery ligation. Previous reduction of the invaginated segments was not attempted. The specimen was exteriorized through periumbilical midline incision, and primary extracorporeal anastomosis was performed using double-layer manual sutures. Gross examination of the specimen revealed a tumor mass of the ileocaecal valve measuring 50 × 45 mm which seemed infiltrate muscular layer (Figure 5).

**Figure 6:**
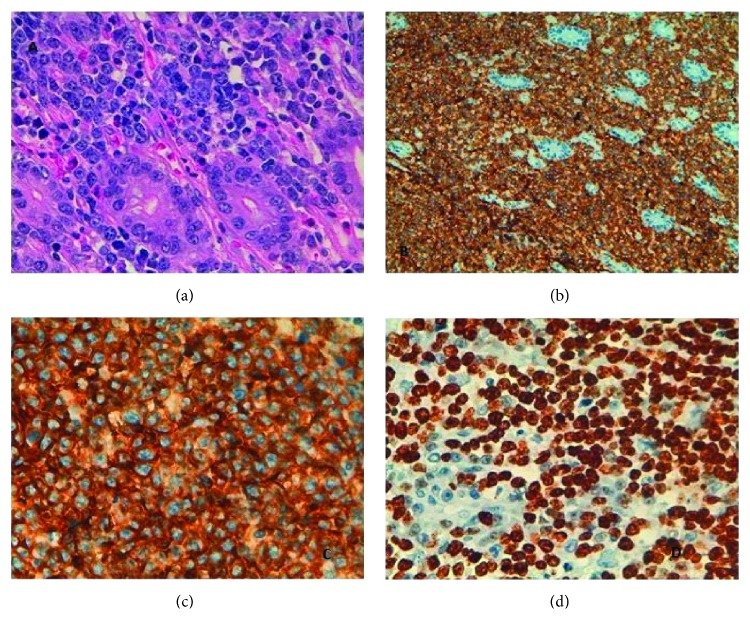
Histological and immunohistological examination of the specimens showing diffuse large B-cell non-Hodgkin's lymphoma. (a) Magnification ×400, HE staining, polymorphic large B-cells with irregular nuclei. (b) Magnification ×400, CD20(+). (c) Magnification ×400, CD79(+). (d) Magnification ×400, Ki67/Mib1 > 95% (high-grade B-cell lymphoma).

Microscopy examination showed ileocaecal valve section presenting dense proliferation of large-sized atypical lymphoid cells with eosinophilic cytoplasm and one or various irregular nucleoli next to the basal membrane. Histopathology of 25 regional and omental lymph nodes revealed focal lymphomatous involvement. Immunophenotypic profile was CD20+, CD79 alfa+, CD10+, BCL2−−+, BCL6−−+, CD5−++, Ciclina D1−, CD3−, CD30−, and ALK−. Proliferation index was high (Ki67/MIB-1 > 95%) (Figure 6). Fluorescent in situ hybridization (FISH) showed typical Burkitt's disease chromosomal translocation: t(8;14)(q24;q32).

Postoperative course was uneventful, and patient was discharged 4 days after surgery. Six weeks after surgery, the patient underwent bone marrow biopsy and full-body CT scan for a further evaluation of the disease. Bone marrow biopsy demonstrated normal proliferation and maturation of all cell lines; CT scan did not show other disease localizations. The patient received a hyper-CVAD combined chemotherapy (cyclophosphamide, doxorubicin, vincristine, and prednisone). At 8-month follow-up before this report, patient is still alive and free of disease.

## 3. Discussion

BL is a B-cell lymphoma with a short doubling time and usually developing an enormous tumoral mass (bulky disease) [2]. BL is characterized by a chromosomal translocation that results in deregulation of the c-MYC oncogene. Burkitt's lymphoma (BL) is a disease usually affecting children and young adults, rarely adults. The first case of Burkitt's lymphoma (BL) was observed in African children by Burkitt in 1958 [3].

Burkitt's lymphoma is more frequent in males rather than females with a ratio of 2 : 1 [4, 5]. In reviews including a considerable number of adult patients with abdominal Burkitt's lymphoma, a percentage from 33% to 50% were more than 20 years old [4, 5].

In endemic areas, BL usually involves the facial bones, particularly the jaw, maxilla, and orbit, especially in young children, associated with Epstein-Barr virus (EBV) infection [6, 7]. Bone marrow involvement is observed in progressive disease [8].

In nonendemic BL, the presence of an abdominal mass is a common finding at the first medical examination. Burkitt's and MALT lymphoma are the most common lymphomas of the small bowel and represent the 42.5% of all lymphomas. American Burkitt's differs from the African type described by Burkitt in 1958 because of the increased propensity for widespread involvement within the abdominal cavity [9].

Intussusception was first described by Barbette of Amsterdam in 1674 [10]. Intussusception in leukemia patients is found almost exclusively in the pediatric population, rarely in adults [11, 12]. In the literature, we found 4 clinical cases of intussusception in adult leukemia patients [11, 13]. In most cases, the leukemic infiltration of the bowel acted like a lead point for the development of the intussusception, even if it is described as a case in which imaging and histological studies have not revealed any leukemic infiltration of the organ [13].

In contrast with children intussusception, a demonstrable etiology is found in 70–95% of cases of adult intussusception and primitive or secondary neoplasms are responsible/the cause of 40% of this condition. Malignancies represent 30% of all small bowel intussusceptions and from 63% up to 68% of large bowel ones [14, 15].

A previous history of chemotherapy and radiotherapy could be potential risk factors for secondary ileocaecal BL [16].

While intussusception in children has a well-known/well-defined clinical presentation for its frequency, intussusception in adults may present with nonspecific symptoms. In Begos et al.' series, 75% of patients presented with obstruction symptoms, 5% with acute abdomen. At physical examination, a palpable abdominal mass was found in a third of cases [17]. The most frequent symptoms are abdominal pain of various character and intensity, vomit, weight loss, permanent fatigue, night perspiration, and sporadic gastrointestinal bleeding. Rarely, complications like acute peritonitis, in case of small bowel perforation, or small bowel obstruction may occur [18].

Laboratory findings are nonspecific in adult intussusception. Transabdominal sonographic study of the bowel intussusception demonstrated an elevated diagnostic accuracy [19], and it is considered a first level diagnostic technique, used directly in the patient's rooms [20]. Sonographically, intussusception appears as a superficial oval formation in longitudinal section with a target shape in transversal section, often with intraperitoneal free fluid [21]. TC scan is a gold standard in ileocolic intussusception diagnosis.

Surgery, chemotherapy, radiotherapy, and radioimmunotherapy are different weapons to fight with GI lymphoma [22].

The role of surgery in GI Burkitt's lymphoma remains controversial. Burkitt's lymphoma responds dramatically to chemotherapies inducing rapid tumor regression and often long-term remission [23]. The surgical treatment in Burkitt's lymphoma intussusception due to localized disease consists in resection and anastomosis of the pathological tract [24]. On the other hand, Magrath et al., considering a study in Burkitt's lymphoma patients in Uganda, suggest that an aggressive operative debulking, with a tumor removal of 90%, performed before the chemotherapy may increase survival [25].

In the emergency settings, there are no evidence-based guidelines leading to which surgical techniques (laparoscopic or laparotomic) to prefer. This choice should be made based on clinical judgment and surgeon's experience. Laparoscopic approach was useful because it allowed us to diagnose the cause of occlusion and resolve it in oncological way considering the risk of an underlying malignancy. Outcomes following laparoscopic colectomy in this setting resulted in reduced length of stay, lower complication rates, and lower costs. Increased adoption of laparoscopy in the nonelective settings should be considered [26]. The laparoscopic approach to acute abdomen, when performed by experienced surgeon, is a feasible alternative to open surgery [27]. Nevertheless, some authors prefer the open technique in colic intussusception, because of the high probability of associated malignant lesions [28].

Thanks to the better understanding of the biology of the disease, for there has been an improvement in chemotherapeutic drugs [29]. The current 5-year survival for advanced Burkitt's lymphoma in children and young adults has increased of 2-3 times in the last 3 decades, from 85% to 90% with less than 6 months of intensive chemotherapy [30].

## 4. Conclusions

Burkitt's lymphoma determining ileocaecal intussusception, although rare in adults, must be considered in intestinal obstruction differential diagnosis, especially when a palpable mass in the right iliac region is present. Imaging studies, particularly abdominal CT, may consent a correct preoperative diagnosis. Definitive treatment and management of intussusception should be individualized according to the patient's age and tumour localization. The surgical approach in GI Burkitt's lymphoma has not yet been coded. In our case, considering intestinal obstruction, early surgical intervention and total tumor removal may be mandatory.

Surgery is always indicated in case of organic obstruction causing the intussusception. Several studies showed that laparoscopic-assisted right colectomy results in less blood loss, a shorter length of hospital stay, and lower postoperative short-term morbidity compared with open resections, also in emergency settings. Minimally invasive surgery should be preferred for abdominal emergencies, like ileocaecal intussusception.
